# The Influence of Tribological Pairings and Other Factors on Migration Patterns of Short Stems in Total Hip Arthroplasty

**DOI:** 10.1155/2017/8756432

**Published:** 2017-04-13

**Authors:** Thilo Floerkemeier, Michael Schwarze, Christof Hurschler, Jens Gronewold, Henning Windhagen, Gabriela von Lewinski, Stefan Budde

**Affiliations:** ^1^Department of Orthopaedic Surgery, Hannover Medical School, Anna-von-Borries-Str. 1-7, 30625 Hannover, Germany; ^2^Laboratory for Biomechanics and Biomaterials, Department of Orthopaedic Surgery, Hannover Medical School, Anna-von-Borries-Str. 1-7, 30625 Hannover, Germany

## Abstract

Over the last decade, the number of short stem total hip arthroplasty procedures has increased. Along with the possible benefits associated with short stems is a smaller implant-bone contact surface, which may have a negative influence on primary stability and impair osseointegration. Previous studies observed migration of short stems, especially within the first three months. The variables that influence migration in short stem hip implants remain unknown. Therefore, the purpose of this study was to associate the migration of short stems with its possible influencing variables. Migration data from two different short stem studies were retrospectively analyzed. Migration within the first two postoperative years was determined by model-based Roentgen stereophotogrammetric analysis. Migration was correlated to bearing couple, type and size of stem, size of acetabular cup, and age, gender, weight, and height of patients using a multiple factor analysis. Eigenvalue analysis explained 80.7% of the overall variance for the first three dimensions. The four most dominant variables in the first dimension were weight, stem size, acetabular cup size, and patient height (correlations of 0.81, 0.80, 0.71, and 0.70, resp.). None of the analyzed parameters (bearing couple, type and size of stem, size of acetabular cup, and age, gender, weight, and height of patients) affected the migration pattern of short stem THA with primary metaphyseal fixation.

## 1. Introduction

Over the last decade, the use of short stems in total hip arthroplasty (THA) has increased. The benefits of short stem arthroplasty include a more physiological load transfer to the proximal femur, resulting in different bone-preserving strategies, as well as a minimally invasive, muscle-protecting implantation technique [1–3]. Because of these advantages, this procedure is especially well suited for younger patients [4, 5]. Manufacturers claim that short stem implants provide a bone-preserving alternative to conventional implants, ensuring better conditions for any necessary revision surgery by reducing the need for bone resection during primary surgery and resulting in less bone loss due to less stress shielding. However, the design of short stems results in a smaller implant-bone contact surface, which may cause inferior primary stability and be associated with higher migration rates compared to traditional stems. This may increase the risk of implant migration and the impairment of osseointegration [6]. Furthermore, femoral neck retention in hip arthroplasty results in an increase in the torsional load-bearing capacity of the proximal femur compared to neck resection [7]. Previous studies of short stem THA have found migration between 0.39 and 1.5 mm within 2 years; the migration typically occurs within the first three months [8–14]. After three months, very little if any migration was observed. However, short stems are very different in their shape and anchoring philosophy and therefore a general migration pattern is not applicable.

Several factors may affect migration patterns after THA. One of these is the choice of the bearing couple. Current standard bearing couples are ceramic-on-ceramic or ceramic-on-polyethylene. Ceramic-on-ceramic THA may stress the implant-bone interface more than a ceramic-polyethylene THA due to lower elasticity of the ceramic that may be assumed to lead to increased transmission of impulses to the implant-bone interface during extreme impacts. The aim of this retrospective study was to determine whether this potentially increased stress causes increased migration of short stems by means of an RSA study and to assess whether the choice of bearing couple affects the migration characteristics. We hypothesize that use of a ceramic-on-ceramic bearing induces higher migration compared to ceramic-on-polyethylene bearings. Furthermore, the influences of other patient- and implant-specific factors such as weight, height, gender, age, and size of the components on migration patterns of short stem THA with primarily metaphyseal anchorage were studied.

## 2. Materials and Methods

### 2.1. Patient Cohort

In this retrospective study (evidence level III), 78 patients were included. The indication for surgery was osteoarthritis of the hip (Kellgren and Lawrence III-IV). This cohort was combined from two different RSA studies: one analyzing patients after implantation of the METHA® system (Braun Aesculap, Tuttlingen, Germany) (60 patients; IRB number 4565, Ethics Committee Hannover Medical School) and the other analyzing patients after implantation of the Nanos® system (OHST Medizintechnik AG, distributed by Smith & Nephew GmbH, Marl, Germany) (18 patients; IRB number 5588, Ethics Committee Hannover Medical School). Both patient cohorts were followed over two years at three, six, twelve and 24 months after surgery. A total of 54 patients were analyzed after 2 years (Table 1). Both cohorts had similar demographic characteristics: the METHA group revealed a mean weight of 79.5 ± 13.3 kg, a mean height of 172 ± 10 cm, and a mean BMI of 26.7 ± 3.6 kg/m^2^, while the Nanos group revealed a mean weight of 78.1 ± 13.3 kg, a mean height of 171 ± 8 cm, and a mean BMI of 26.6 ± 3.3 kg/m^2^. The implanted stem size ranged from 1 to 8 and cup size ranged from 46 to 60; the liner material was PE in 20 patients and ceramic in 34 patients after 2 years (Table 1).

Inclusion criteria for the primary THA performed were age between 30 and 75 years at date of surgery and at least three months between surgical procedures in the case of bilateral THA. The following exclusion criteria were defined as mentioned previously [10]: previous surgery on the affected joint except for arthroscopic surgery, local or systemic infections, previously diagnosed osteoporosis, pronounced coxa valga with a femoral neck angle of >145°, pronounced coxa vara with a femoral neck angle of <125°, insufficient femoral or acetabular bone stock or indication for a revision cup (as determined by the surgeon's assessment of the preoperative radiographs), neurological or spinal disease with neurological movement disorders, alcoholism or addictive disorders, American Society of Anesthesiologists (ASA) score of 3 or 4, BMI > 30, pregnancy, allergy to elements of the implanted device, and insufficient command of the language to understand patient information and consent.

### 2.2. Surgical Procedure and Implants

#### 2.2.1. Nanos

**Figure 1:**
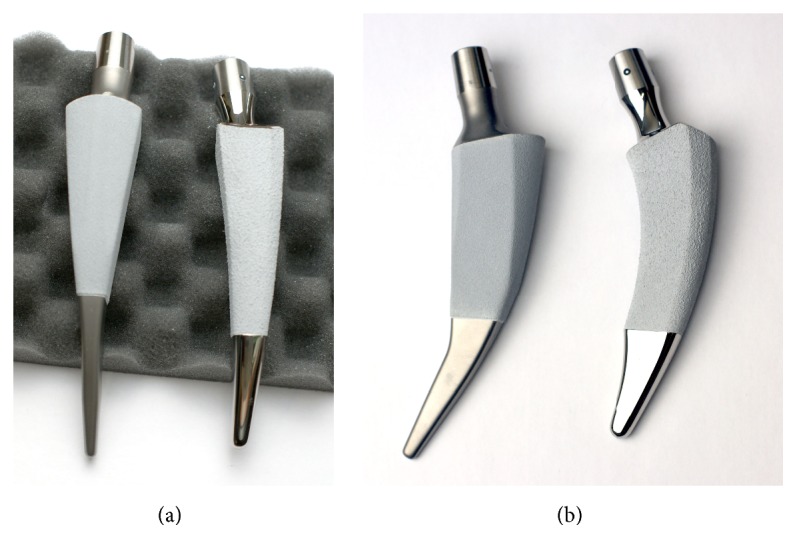
Comparison of two implants (a) in lateral view and (b) in frontal view. METHA (left in each image) and Nanos (right in each image) implants.

**Figure 2:**
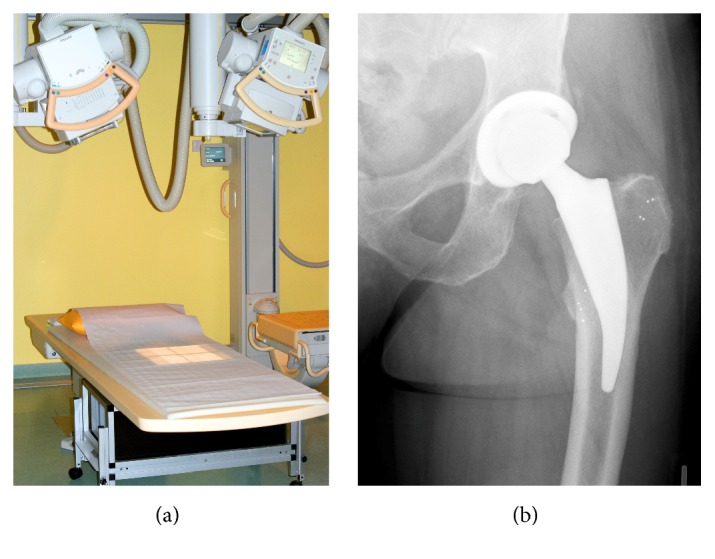
Uniplanar RSA measurement setup. (a) The X-ray tubes were each oriented at a 20° angle to the horizontal and the film to focus distance was 160 cm. The carbon calibration box was under the examination table. (b) Implant migration was calculated by rigid body kinematics using up to ten tantalum beads, which were inserted into the bone surrounding the implant and serving as fixed reference.

The Nanos stem (OHST Medizintechnik AG, distributed by Smith & Nephew GmbH, Marl, Germany) with a choice of different sizes (sizes 2–8) and the EP-FIT PLUS™ acetabular cup (Smith & Nephew) were implanted. The Nanos short stem is made of a plasma-coated titanium forged alloy (Ti6Al4V) with a proximal calcium phosphate coating and a polished tip (Figures 1(a) and 1(b)). After preparation of the stem and just before implantation of the femoral component, at least 5 tantalum beads with a diameter of 1 mm (Tilly Medical Products AB, Lund, Sweden) were implanted in the regions of the greater and lesser trochanters (Figure 2(b)). Each implantation was performed by the same senior surgeon. Postoperative physiotherapeutic treatment allowed full weight bearing immediately after surgery.

#### 2.2.2. METHA

For the cohort of patients with implanted METHA stem (B Braun Aesculap, Tuttlingen, Germany), all surgeries included the implantation of an acetabular component (threaded or press-fit cup (B Braun Aesculap, Tuttlingen, Germany)). Each surgery was performed by one of four senior surgeons. The METHA short stem was used as the femoral component (size 0–7 with different CCD angles (130°, 135°, or 140°)). The METHA stem is a cementless, collarless, and tapered prosthetic stem that is anchored metaphysically within the closed ring of the femoral neck (Figures 1(a) and 1(b)). For osseointegration, the METHA arthroplasty is coated with Plasmapore®, a *μ*-calcium phosphate layer. This layer is purported to have an osteoconductive effect that accelerates contact between the bone and the prosthetic stem.

In both patient cohorts, the femoral ball head material was 4th-generation ceramic (Biolox® Delta, CeramTec, Plochingen, Germany). The choice of the material for the acetabular insert (either ultrahigh-molecular-weight-polyethylene according to ISO 5834-2 or Biolox Delta ceramic) was made in an individual decision making with informed consent of the patient depending on age and expected postoperative activity level. Polyethylene was preferably used in older patients with lower activity levels whereas ceramic was rather used in younger and more active patients in order to avoid abrasive wear.

### 2.3. RSA Setup

The RSA setup used has been previously described by Budde et al. [10]. Patients were followed using a MBRSA method to detect implant migration [15, 16]. All RSA measurements and migration calculations were performed according to ISO 16087:2013(E) and the RSA standardization guidelines [17] including double examinations to confirm the precision of the method (Table 2). For each short stem size implanted, reverse-engineering (RE) surface models were created by a structured light scanner (ATOS II, GOM GmbH, Braunschweig, Germany). Patients were positioned in standard supine position on a flat table with the calibration box directly under the examined area of interest (Figure 2(a)). Reference RSA radiographs were taken 2 to 9 days after surgery and follow-up investigations were performed at 3, 6, 12, and 24 months. Implant migration is presented as the resulting total migration, defined as $\sqrt{x^{2}+y^{2}+z^{2}}$.

### 2.4. Statistics

The influence of several contributors on migration was analyzed by an exploratory multiple factor analysis with the following variables: acetabular insert material, stem type and size, acetabular cup size, and age, gender, weight, and height of patients as well as migration. The FactoMineR package (Version 1.28) for the R statistical software (Version 3.0.2, The R Foundation for Statistical Computing) was used to conduct the analysis [18]. Data at the 24-month follow-up were used for analysis. In order to compare results of subsets of the data between single factors, the Wilcoxon-Mann-Whitney test was used. The significance level alpha was set to 0.05 for all comparisons.

## 3. Results

**Figure 3:**
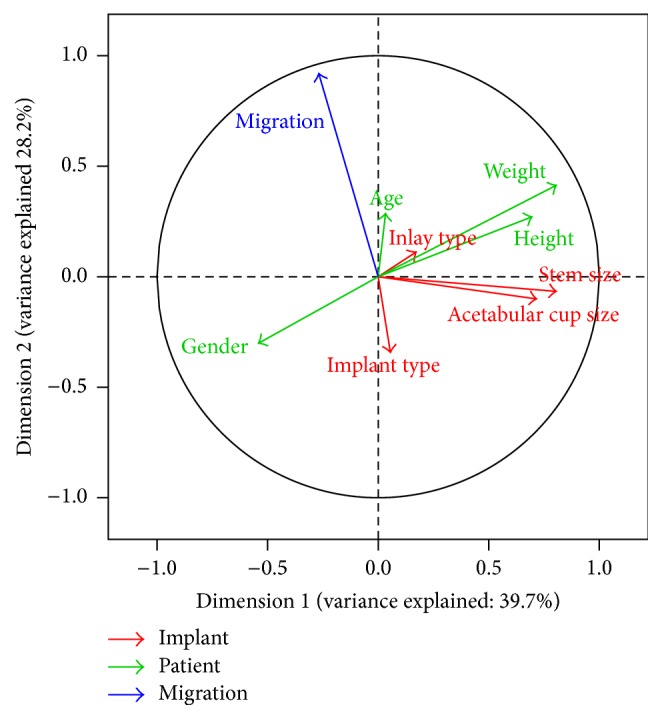
Correlation circle of the first two dimensions. Variables attributed to the patient are shown in green, variables of implants are shown in red, and migration is shown in blue.

The Kaiser-Meyer-Olkin analysis revealed the adequacy of the sampling (KMO = 0.69, above the accepted threshold of 0.5) [19]. Bartlett's test of sphericity supported the sufficient correlation between variables for a factor analysis (chi-square = 154, *p* < 0.001). Eigenvalue analysis explained 80.7% of the overall variance for the first three dimensions. The four most dominant variables in the first dimension were weight, stem size, acetabular cup size, and patient height (correlations of 0.81, 0.80, 0.71, and 0.70, resp.; Figure 3). The second dimension was dominated by implant migration, patient weight, and type of implant (correlations of 0.92, 0.41, and −0.34, resp.). The third dimension was determined by age and acetabular cup size (correlations of −0.61 and 0.38, resp.). Implant migration was mostly negatively correlated with implant type.

**Figure 4:**
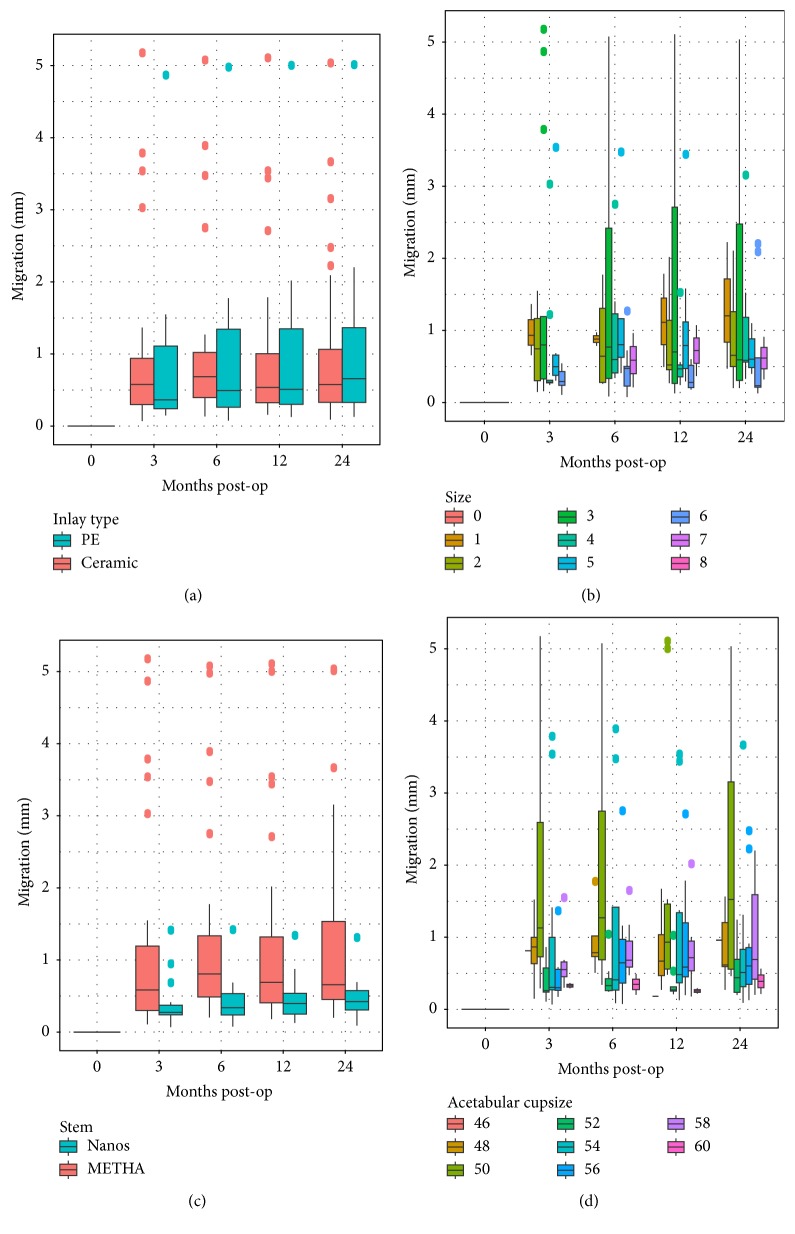
Boxplots of resulting stem migration grouped according to implant-related factors: (a) insert type, (b) stem size, (c) stem type, and (d) acetabular cup size.

**Figure 5:**
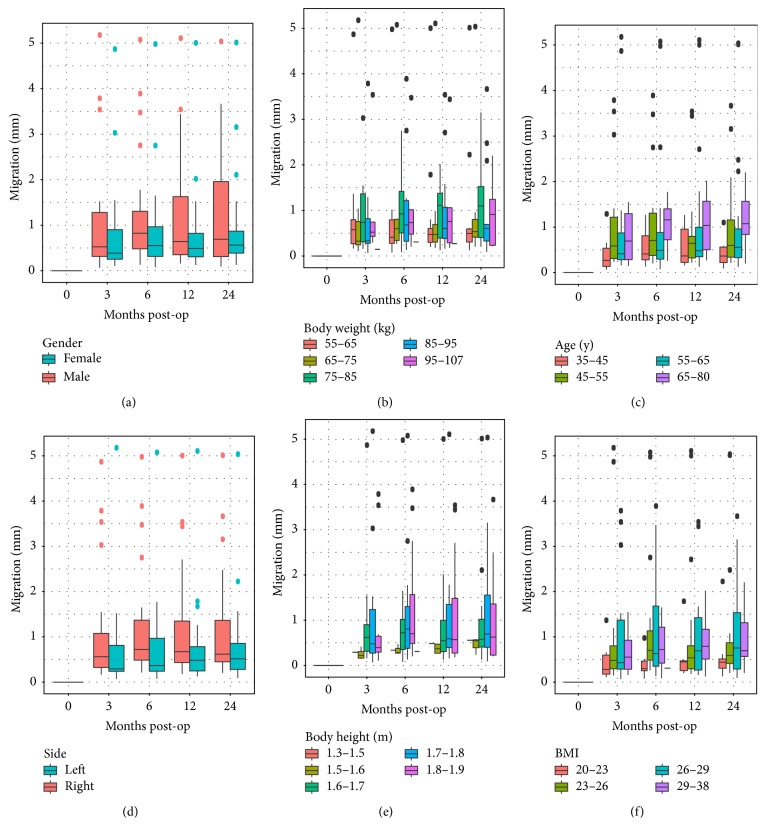
Boxplots of resulting implant migration dependent on follow-up and different patient-related factors: (a) gender, (b) body weight, (c) age, (d) operated side, (e) body height, and (f) BMI.

By analyzing migration plotted against each of the possible influencing factors, a high variability within each group within a factor can be observed (Figures 4 and 5). An influence on migration can be observed for implant type, acetabular cup size, gender, and implantation side. Specifically, the acetabular insert material had practically no influence on migration or the migration pattern.

## 4. Discussion

The aim of the current study was to use RSA to determine whether the choice of the bearing couple affects the migration characteristics of short stem THA and whether other factors influence the short stem migration. We hypothesized that the use of ceramic-on-ceramic bearing induces higher migration compared to ceramic-on-polyethylene bearing because of the higher mechanical stiffness that results in increased transmission of impulses to the implant-bone interface during impacts. In this analysis of the prospectively gathered RSA data two years after implantation of a short stem THA (Nanos or METHA) using either ceramic-on-ceramic or ceramic-on-polyethylene, we did not observe any influence of the bearing couple on the short stem migration patterns.

The clinical relevance of implant migration has been reported in several studies [20, 21]. It has also been shown that the migration pattern over time is by far more important with regard to implant survival than is the absolute value of the total migration itself. Very little data exist regarding the factors that influence early (less than six months) implant migration. Therefore, we analyzed several patient- and implant-specific factors, including the bearing couple, with regard to their influence on implant migration. In particular, this is the first study to analyze the factors influencing implant migration in partial neck-preserving short stem THAs.

Furthermore, other factors, including age, height, weight, gender, or size of the components, did not affect the migration pattern of the partial neck-preserving stems. However, the factor analysis revealed a correlation of migration with implant type. Indeed, the Nanos migrated significantly less than the METHA (*p* = 0.012). However, it is well known from a variety of other RSA studies that different implant designs show substantial differences regarding the absolute values of migration due to different concepts of implant anchorage [22].

There was also a weak correlation of migration with age, but this might be confounded by the younger average age in the Nanos group (mean age Nanos: 53 y; METHA 59 y). The reason for increased migration therefore is likely the use of METHA rather than advanced age.

Factors that correlate very well with each other include weight and height as well as stem and acetabular cup size. This is not surprising, as the size of bony structures and therefore the required size of the implants are directly related to the height and, to some degree, the weight of a patient. Furthermore, gender is negatively correlated with those four variables. This is a consequence of women being physically smaller than men in our cohort.

The migration of ceramic-on-ceramic components was similar to ceramic-on-polyethylene after THA. This refutes our hypothesis that the mechanical properties of the ceramic-on-ceramic bearing may lead to higher stresses at the implant-bone-surface during impacts: we assumed that these hypothesized stresses may lead to higher micromotion and thus impairment of osseointegration that may in turn be detected by higher migration measured by RSA. The modulus of elasticity of Biolox Delta ceramic reported by the manufacturer (358 GPa) is more than 600 times higher than that of cross-linked polyethylene (532 MPa) [23]. It remains unclear whether impacts are either already absorbed somewhere on their way to the implant-bone-surface (e.g., in the head-conus junction or in the femoral stem itself) or whether they are indeed transmitted to the implant-bone-surface but simply do not evoke negative effects there.

A similar conclusion with regard to the acetabular cup was drawn by Zhou et al., who compared ceramic-on-ceramic with metal-on-polyethylene combinations for migration pattern in 61 patients and did not observe any differences in cup migration between the groups [24]. Other RSA studies analyzing the influence of bearing couples on migration patterns have not been performed.

Ochs et al. conducted a prospective randomized EBRA-study (Ein-Bild-Roentgen-Analyse-femoral component analysis) to determine the influence of either ceramic-on-ceramic or ceramic-on-polyethylene bearings on migration [25]. They found that primary subsidence was independent of the chosen bearing couple.

Furthermore, it was hypothesized that bone quality and factors possibly influencing bone quality such as age, gender, and bone mineral density (BMD) may affect the migration pattern of THA. Poor bone quality can be judged by DXA-scans to measure BMD [26]. It could be assumed that weaker bone structure in terms of osteoporosis may induce a greater migration within the first months after implantation of a short stem. In the current study, the influences of age and gender on the migration pattern were analyzed. Our data revealed only marginal effect on the migration pattern of the short stems. However, the inclusion criteria for implantation of a short stem THA were an age of less than 70 years. Thus, most patients participating in this study were likely too young to have poor bone quality due to osteoporosis, although DXA-scans were not performed.

Furthermore, it can be speculated that higher BMI may induce increased migration due to a greater load transmitted to the proximal femur and that the greater the stress conducted by the THA to the femur, the greater the risk of subsidence. However, the data of the current study revealed that weight did not affect the migration of the stems. This confirms previous findings such as those of Freitag et al., who analyzed the migration pattern of 72 femoral short stem prostheses using EBRA-FCA and found a mean axial subsidence of 1 mm (±1.4 mm) after 24 months [12]. According to their results, BMI, gender, and implant offset did not influence migration on a statistically significant level, although a tendency towards more migration in obese and female patients was observed. Stihsen et al. analyzed the subsidence of the Vision 2000 (Depuy) depending on body weight and BMI using EBRA-FCA [27]. They observed that physical characteristics such as body weight and height had significant influence on migration patterns of this cementless femoral component.

Finally, we questioned whether the size of the femoral stem affects the migration pattern. In clinical practice, we considered a coxa magna as a contraindication for short stems with primary metaphyseal anchorage. On the other hand, our clinical experience with cases of small femoral canals and especially dysplastic femoral necks is that the stems interlock tightly without a change of subsidence. Therefore, we hypothesized that larger stem sizes may induce insufficient press fit within the proximal femur and lead to an increased migration pattern. However, our data did not show an increase in migration for larger femoral components.

The advantage of this study was the use of the reliable RSA method for migration analysis. In addition to total migration, the rate of migration over the time is an important criterion for the prediction of later stability. RSA allows the precise measurement of early implant migration, which has been shown to correlate with later aseptic implant loosening [20, 28]. The RSA method is considered the gold standard for the in vivo assessment of implant fixation [29, 30].

An advantage and simultaneous disadvantage of this study are that the study cohort was assembled from patients after implantation of a METHA short stem and 18 patients after implantation of a Nanos short stem. The benefit is the large number of patients and that the analysis was based on more than one implant, ensuring that the observations made in this study are not derived from characteristics of only one particular implant. On the other hand, the implant design has a significant influence on implant migration and may have biased our results although the statistical methodology was chosen in order to subtract this bias as much as possible. Another potential source of bias is that the choice of the bearing couple was made depending on age and expected postoperative activity level, leading to a slightly lower age of patients treated with ceramic-on-ceramic bearings. Since age showed a weak correlation with migration, it may be assumed that better bone quality in younger patients may have counteracted a potential effect of ceramic-on-ceramic bearings on migration. However, it is most likely that the correlation of age and migration was rather confounded by the implant type as mentioned above.

A weakness of the study protocol is that the postoperative RSA examinations were not performed immediately but after a mean period of 2–9 days postoperatively. Because full weight bearing was allowed, some stems might have already migrated before the first RSA examination. However, as could be shown in other studies, the postoperative load-bearing regimen does not influence the migration [31, 32].

In summary, none of the analyzed parameters dominantly affected either the absolute migration or the migration pattern of short stem THA. The tested devices demonstrate sufficient anchorage to achieve primary stability, and this stability is achieved regardless of the presence of suspected influencing factors.

## Figures and Tables

**Table:** (a) Implant variables

Type (*n*)	Acetabular cup size (*n*)	Acetabular insert material (*n*)
METHA: 40 Nanos: 14	size 46: 1 size 48: 5 size 50: 9 size 52: 9 size 54: 11 size 56: 10 size 58: 7 size 60: 2	*Polyethylene*: 20 (total) 15 (METHA-stem) 5 (Nanos-stem) *Ceramic*: 34 (total) 25 (METHA-stem) 9 (Nanos-stem)

**Table:** (b) Patient variables

Age (y)	Sex (*n*)	Weight (kg)	Height (cm)
Median: 58 (range: 36–72)	Male: 22 female: 32	Median: 80 (range: 56–107)	Median: 172 (range: 140–190)

**Table 2:** RSA parameters and equipment used during image acquisition and analysis.

Parameter	Value
Precision of arrangement:	Valid for METHA
Translation (*Tx*, *Ty*, *Tz*)	*Tx*: 0.014 ± 0.086, *Ty*: −0.019 ± 0.147, *Tz*: −0.046 ± 0.229
Rotation (*Rx*, *Ry*, *Rz*)	*Rx*: 0.03 ± 0.77, *Ry*: 0.09 ± 3.12, *Rz*: −0.01 ± 0.21
Calibration cage	Carbon Box Leiden 10 Hannover
X-ray tubes	2x SRO3310 ROT 360 (Philips)
Tube voltage/current	90 kV/12.5 mAs
Angle between X-ray paths	40 deg
X-ray cassette	36 × 43 cm IP Cassette Type CC (Fuji)
Cassette digitizer	PCR Eleva Corado (Philips), resolution: 125 dpi
Condition number cut-off threshold	120
Mean rigid body error threshold	0.35 mm
RSA software version	Medis Specials Model Based RSA 3.2/3.31
